# The Clinician-Scientist in Vision Science: A Rare and Endangered Species

**DOI:** 10.1167/tvst.10.1.30

**Published:** 2021-01-21

**Authors:** Rajesh C. Rao

**Affiliations:** 1 Departments of Ophthalmology & Visual Sciences and Pathology, University of Michigan, Ann Arbor, MI, USA. e-mail: rajeshr@med.umich.edu

I thank Dr. Van Gelder for his editorial^1^ on our recent article published in *TVST*.^2^ Dr. Van Gelder is one of the most accomplished physician–scientists in ophthalmology, and his stature and experience lend much-needed attention to the leaky clinician–scientist career pipeline. Although I agree with nearly every point in the editorial, I want to correct a statement in that piece regarding an exclusion in our study of clinician–scientists who obtained their second R01 (new or renewal) outside of the National Eye Institute (NEI). In fact, we did analyze second R01 grants awarded to clinician–scientists that were funded outside the NEI. Indeed, we found that, of those clinician–scientists who obtained a second R01, ∼10% obtained R01 grants funded from a non-NEI National Institutes of Health Institute or Center, such as the National Institute of General Medical Sciences and the National Cancer Institute. Our data highlight the diversity of scholarship conducted by R01-funded clinicians in ophthalmology and optometry. Our findings further suggest that future efforts supporting interdisciplinary efforts may be part of a strategy to sustain independent careers in vision research.
